# De Novo Assembly, Characterization, and Comparative Transcriptome Analysis of Mature Male and Female Gonads of Rabbitfish (*Siganus oramin*) (Bloch & Schneider, 1801)

**DOI:** 10.3390/ani14091346

**Published:** 2024-04-30

**Authors:** Xiaolin Huang, Zhong Huang, Qiang Li, Wenjun Li, Chong Han, Yukai Yang, Heizhao Lin, Qiaer Wu, Yanbo Zhou

**Affiliations:** 1 Key Laboratory of South China Sea Fishery Resources Exploitation & Utilization, Ministry of Agriculture, South China Sea Fisheries Research Institute, Chinese Academy of Fishery Sciences, Guangzhou 510300, Chinalinheizhao@163.com (H.L.); 2 National Fishery Resources and Environment Dapeng Observation and Experimental Station, Shenzhen Base of South China Sea Fisheries Research Institute, Chinese Academy of Fishery Sciences, Shenzhen 518121, China; 3 School of Life Sciences, Guangzhou University, Guangzhou 510006, China

**Keywords:** rabbitfish, transcriptome, gonad, reproduction-related genes

## Abstract

**Simple Summary:**

*Siganus oramin*, as a commercial species, is a well-received table fish in southeastern China. However, valuable reproduction-related data are still scarce. In the present study, gonad transcriptome analysis was first performed using Illumina Novaseq technology. Comparative transcriptome analysis of adult male and female gonads identified a large number of differentially expressed genes. Among them, many reproduction-related genes that play important roles in gonad differentiation and development were found. These data may contribute greatly to the regulation of rabbitfish reproduction.

**Abstract:**

The rabbitfish, *Siganus oramin*, is a commercially important table fish in southeastern China. However, there have been few studies on its gonad development and reproduction regulation. Comparative transcriptome analysis was first performed on adult male and female gonads of *S. oramin*. In total, 47,070 unigenes were successfully assembled and 22,737 unigenes were successfully annotated. Through comparative transcriptome analysis of male and female gonads, a total of 6722 differentially expressed genes were successfully identified, with 3528 upregulated genes and 3154 downregulated genes in the testes. In addition, 39 differentially expressed reproduction-related genes were identified. Finally, quantitative real-time PCR was used to validate the expression levels of several differentially expressed genes. These results provide important data for further studying the function of reproduction-related genes and the molecular mechanism regulating gonad development and reproduction in *S. oramin*.

## 1. Introduction

The rabbitfish (*Siganus oramin*) is broadly distributed in coastal coral reef and shallow water areas of tropical and subtropical zones. It is a kind of small omnivorous fish (weight: 104.12–193.46 g) that belongs to family Siganidae and order Perciformes, and its annual output is about 10,000 tons. It is a well-received table fish species in south and east China due to its high nutritional value and good taste [1]. It has adapted well to offshore areas and often gathers in groups in offshore seaweed cultures or cage cultures. In wild environments, it mainly feeds on residual bait from aquaculture and algae attached to rocks, cages, and other solid supports [2]. In addition, the ovary development of rabbitfish is completely synchronous, and the breeding period ranges from late March to early May [2]. Despite its strong adaptability, the development of rabbitfish aquaculture is very slow. Currently, most seedlings of rabbitfish come from marine fishing.

Previous studies mainly focused on gut microbiota [3], feed [4], immunity [5], and toxicity [6,7], with less reproduction-related research. However, gonad development and reproduction regulation are critically important for the regulation of aquaculture. Moreover, as lower vertebrates, the reproduction process of fish is often regulated by many complex factors, such as genetic and environmental factors [8]. Thus, gonad differentiation and development are usually different among different species. For rabbitfish, our previous research found that the testes mature faster than the ovaries and that their period of sexual maturity is very short (around May each year) [2], which increases the difficulty of reproduction regulation and seeding. In fact, in mandarin fish (*Siniperca chuatsi*), mature testes and ovaries can hold for several months spanning April and June [9]. Thus, it is necessary to obtain knowledge of its gonad development, differentiation, and reproduction regulation. However, up to now, nearly no reproduction-related genes have been uncovered for rabbitfish, which has further increased the difficulty of regulating its reproduction.

Recently, next-generation sequencing technology has rapidly developed, and transcriptome sequencing technology has been increasingly applied in studies related to fish sex and reproduction. Based on transcriptome sequencing technology, many reproduction-related genes have been uncovered in a large number of fish species, such as yellow catfish (*Pleteobagrus fulvidraco*) [10], spotted knifejaw (*Oplegnathus punctatus*) [11], Chinese tongue sole (*Cynoglossus semilaevis*) [12], silver sillago (*Sillago sihama*) [13], spot-fin porcupine fish (*Diodon hystrix*) [14], spotted scat (*Scatophagus argus*) [15], mandarin fish (*Siniperca chuatsi*) [16], army fish (*Spinibarbus hollandi*) [17], white head mandarin fish (*Coreoperca whiteheadi*) [18], and so on. Based on these reproduction-related genes, researchers can explore their expression and function more pertinently. In addition, gene expression can reflect information related to gonad development, and farmers could choose the right time to strengthen nutrition for the maturation of the gonads. These reproduction-related genes may contribute greatly to further research on gonad development and reproduction regulation.

In the current study, for the first time, gonad transcriptome sequencing of mature female and male gonads of *S. oramin* was performed. Through assembly and annotation, many functional genes were identified and annotated. Moreover, based on comparative transcriptome analysis, many sex- or reproduction-related genes were uncovered. These reproduction-related genes provide important clues to further explore reproduction regulation in rabbitfish. Our results enrich the current genomic and transcriptomic resources of *S. oramin* and may contribute greatly to further research on its gonad development, differentiation, and reproduction regulation.

## 2. Materials and Methods

### 2.1. Sample Collection

According to previous similar studies [15,17,18], a total of six mature *S. oramin* (about one year old) were obtained before spawning (three males and three females) from the waters of Daya Bay, Guangdong Province, in China in May 2023. The temperature of the environment ranged from 27 to 33 °C, with humidity of about 70–80%. Their lengths ranged from 13 cm to 15 cm with weights from 80 g to 120 g. These fish were first anesthetized using MS-222 (live fish transport anesthetic, Sigma, Saint Louis, MO, USA). Then, the phenotypic sex of each fish was determined by assessing its gonad morphology. Finally, the gonads were rapidly placed in liquid nitrogen within 60 s of sacrifice. All animal processes strictly followed the protocols of the Experimental Animal Ethical Committee of the South China Sea Fisheries Research Institute.

### 2.2. RNA Extraction and Library Construction

Following the manufacturer’s instructions, total gonad RNA was extracted using FreeZol Reagent (Vazyme, Nanjing, China). The concentration and purity of the RNA were preliminarily detected by a Nanodrop 2000 (Thermo Scientific, Waltham, MA, USA). RNA integrity detection was performed by an Agilent 4200 Bioanalyzer (Agilent Technologies, Santa Clara, CA, USA). The qualified RNA was applied to construct transcriptome libraries according to an NEBNext^®^ Ultra™ RNA Library Prep Kit for Illumina^®^ (NEB, Ipswich, MA, USA). The workflow was as follows: the gonad mRNA of *S. oramin* was enriched by magnetic beads containing Oligo (dT); the mRNA was randomly fragmented in a high-temperature environment with metal ions, and the first cDNA strand was synthesized using random hexamers; the enzyme, buffer solution, and dNTPs including dATP, dTTP, dGTP, and dCTP were added to synthesize the complementary cDNA strand; the double-stranded cDNA was purified by magnetic beads; the end was repaired; and an A base was added, followed by ligation of sequencing adapters and fragment size sorting. Using PCR amplification, the sorted fragments were enriched, and the PCR products were purified to form the final sequencing library.

### 2.3. Library Sequencing, De Novo Assembly, and Annotation

Using the Illumina Novaseq6000 (Illumina, San Diego, CA, USA) high-throughput sequencing platform, the obtained cDNA libraries were sequenced according to the Pair-End 150 (PE150) sequencing strategy. For each library, the sequencing data were larger than 6 Gb. The Illumina high-throughput sequencing results were initially presented as raw image data, which needed to be converted into raw reads by Base Calling using CASAVA software version 1.8. Then, Fastp v0.20.1 software was applied to identify and discard splice sequences and low-quality sequences to generate high-quality clean reads using the parameters “-l 150 -q 20” [19]. Based on the clean reads, F1 sequencing data were selected to perform de novo assembly. The Trinity platform was used to assemble the transcriptome sequencing data through Inchworm, Chrysalis, and Butterfly [20]. Then, BUSCO v5.2.2 was applied to evaluate the integrity of the transcriptome assembly [21].

To predict the protein-coding frames of these unigenes, three forward and three reverse reading frames, namely six coding modes, were applied. Then, the non-redundant protein sequence (https://www.ncbi.nlm.nih.gov/, accessed on 10 January 2024) and the Uniprot protein databases (https://www.uniprot.org/, accessed on 20 January 2024) were used to annotate the obtained protein-coding sequences. Finally, the coding mode with the maximum score was considered the final coding mode of the unigene.

In addition, these unigenes were annotated by five major public databases, including Nr, the Kyoto Encyclopedia of Genes and Genomes (KEGG, http://www.genome.jp/kegg, accessed on 18 July 2023), Uniprot, the Clusters of eukaryotic Orthologous Group database (KOG, http://www.ncbi.nlm.nih.gov/COG/, accessed on 19 June 2023), and Gene Ontology (GO, http://geneontology.org, accessed on 17 July 2023), based on homology searches by BLASTP.

### 2.4. Identification of Differentially Expressed Genes (DEGs) and Enrichment Analysis

The Trinity perl command Align_and_estimate_abundance.pl, the Bowtie2 v2.4.2 alignment method [22], and the RSEM (version 1.2.7) estimation method [23] were applied to compare the sequencing data and unigene sequences and to calculate the unigene expression levels. The transcripts per million (TPM) and fragments per kilobase of exon model per million mapped fragments (FPKM) values of each transcript were further calculated by stringtie v1.3.3b [24]. Then, the Python program prepDE.py was applied to convert the stringtie results into a format that could be identified by the “edgeR” package (V3.6) [25,26]. The differential gene expression analysis was carried out using edgeR. |log2FC| > 2 and *p* value < 0.05 were set as the screening conditions for significant DEGs. Finally, according to these DEGs, the significant GO terms and KEGG pathways (*p* value < 0.05) were further enriched by the clusterProfiler program in the R version 4.3.0 package after Fisher’s exact test and the Benjamini correction [27].

### 2.5. Validation of DEGs by Quantitative Real-Time PCR (qRT-PCR)

Using qRT-PCR analysis, fifteen sex-related DEGs were applied to validate the expression levels of the DEGs identified in the transcriptome data. The primers of these DEGs and β-actin (internal reference) were mainly designed by primer 5.0 software (Table 1). First, the cDNA templates were reverse-transcribed by PowerScript RT SuperMix (+gDNA wiper) (GDSBIO, Guangzhou, China). Then, qRT-PCR amplifications were performed using SYBR Green qPCR Mix (GDSBIO, Guangzhou, China) on a Roche LightCycler 480 real-time PCR system. All reactions were performed using the following process: pre-denaturation at 95 °C for 3 min; 40 cycles of 95 °C for 10 s, 60 °C for 20 s, and 72 °C for 15 s; extension at 72 °C for 5 min; and a final dissociation curve process. Three technical replicates were performed for each reaction, and the relative expression was normalized by β-actin using the 2^−ΔΔCT^ method [28].

## 3. Results

### 3.1. Illumina Sequencing and Assembly Results

**Figure 1:**
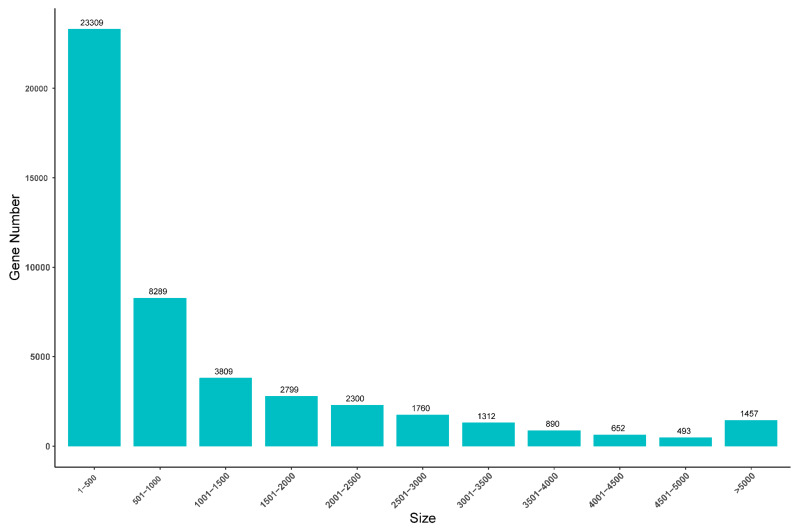
Unigene length distribution of the *S. oramin* gonad transcriptome.

A total of 46.73 Gb of data were generated for six cDNA libraries (7.79 Gb per sample) including three ovaries and three testes, with a mean of 51,920,597 reads per sample. Moreover, the average GC content was 51.47%, Q20 was more than 95%, and Q30 was more than 90% (Table 2). These data were further filtered and assembled, and in total 71,435 transcripts and 47,070 unigenes were successfully obtained. The average lengths of the transcripts and unigenes were 1524 and 1164 bp, respectively. And the average N50 values were 2875 bp and 2488 bp, respectively (Table 3). Moreover, the predicted lengths of most unigenes were >500 bp (49.52%), while only 1457 (3.09%) unigenes were >5000 bp in length (Figure 1).

### 3.2. Unigene Annotation

**Figure 2:**
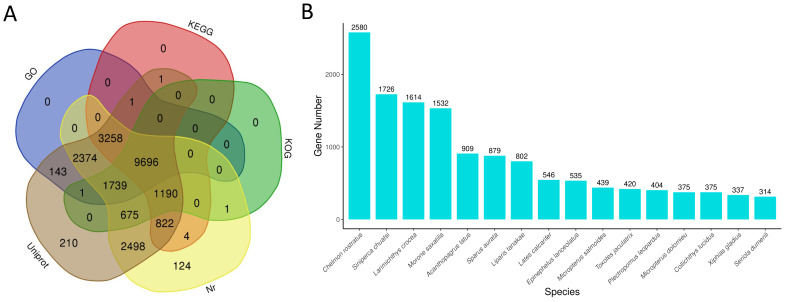
Venn diagram of the functional annotation against five major databases (**A**) and the species distribution according to the results of the Nr annotation (**B**).

All 47,070 unigenes were aligned to five major public molecular databases. However, only 22,737 (48.30%) unigenes were successfully annotated. Nearly all annotated unigenes were successfully aligned to the Uniprot database (22,608; 99.43%) and the Nr database (22,381; 98.43%), while only half of the annotated genes were annotated in the KOG database (13,302; 58.50%) (Figure 2A). Meanwhile, according to the Nr annotation, the BLASTx top-hit species distribution statistics were also estimated. And the results showed that *Chelmon rostratus* (2580, 11.53%), *Siniperca chuatsi* (1726, 7.71%), *Larimichthys crocea* (1614, 7.21%), and *Morone saxatilis* (1532, 6.85%) were the top four species (Figure 2B).

**Figure 3:**
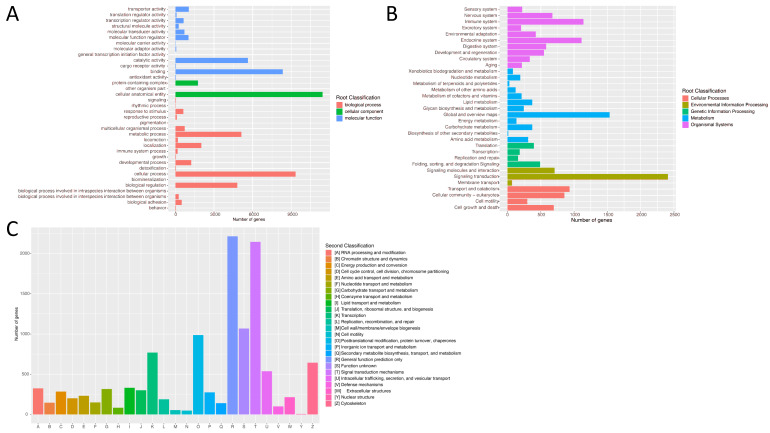
Functional classification of genes according to GO (**A**), KEGG (**B**), and KOG (**C**) databases.

In addition, functional prediction and classification were also assigned to these annotated unigenes based on the GO, KOG, and KEGG databases. According to the GO annotation, a total of 17,212 unigenes were successfully annotated and further categorized into three functional categories, including biological processes, molecular functions, and cellular components. Among these categories, the terms “cellular anatomical entity” in cellular components (11,426 unigenes), “cellular process” in biological processes (9337 unigenes), and “binding” in molecular functions (8331 unigenes) were the most dominant (Figure 3A).

Based on the KEGG annotation, a total of 14,972 unigenes were categorized into five functional categories, including genetic information processing, cellular processes, environmental information processing, metabolism, and organismal systems. The top three secondary categories were “signal transduction” (2408 unigenes), “global and overview maps” (1533 unigenes), and “immune system” (1140 unigenes) (Figure 3B).

In addition, the KOG annotation revealed that 13,320 unigenes were categorized into twenty-five classifications. Among them, “general function prediction only” (2214 unigenes) had the largest distribution, “signal transduction mechanisms” (2145 unigenes) had the second largest distribution, and “nuclear structure” had the smallest distribution (6 unigenes) (Figure 3C).

### 3.3. Differential Expression Analysis

**Figure 4:**
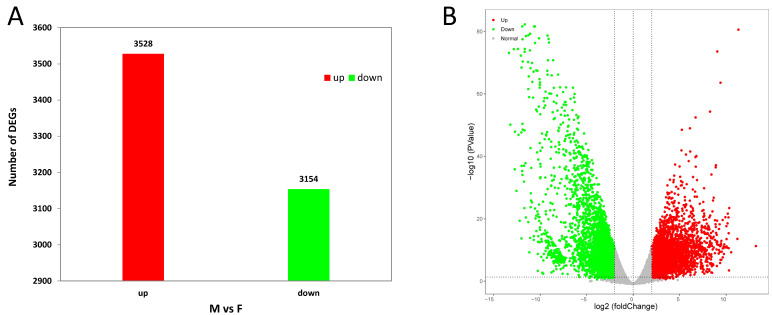
Number of DEGs (**A**) and volcano plot of DEGs (**B**) in male versus female gonads of rabbitfish. Red and green dots represent upregulated and downregulated genes in males.

**Figure 5:**
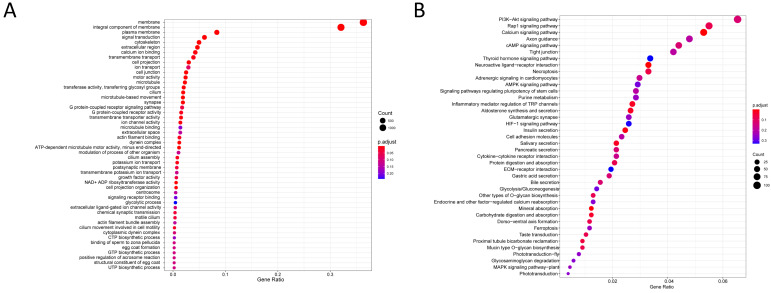
Top 50 GO classifications (**A**) and 40 KEGG pathways (**B**). The dot size represents the number of genes aligned to the GO term or KEGG pathway. The colors, from red to blue, represent the significance of the enrichment.

The gene expression levels in the testes and ovaries were calculated based on the FPKM values. In total, 3528 upregulated genes and 3154 downregulated genes were found in the testes (Figure 4A). All DEGs with a |log2FC| > 2 and a *p* value < 0.05 are visually displayed in the volcano plot (Figure 4B). In addition, the top 50 GO classifications and 40 KEGG pathways are shown in Figure 5. Among the KEGG pathways, the significantly enriched pathways included the calcium signaling pathway, the neuroactive ligand–receptor interaction, mineral absorption, insulin secretion, aldosterone synthesis and secretion, salivary secretion, inflammatory mediator regulation of TRP channels, carbohydrate digestion and absorption, and proximal tubule bicarbonate reclamation (Figure 5B).

In addition, many sex- or reproduction-related genes were further identified among these DEGs (Table 4). Genes such as anti-Mullerian hormone (*amh*), anti-Mullerian hormone type-2 receptor (*amhrII*), bone morphogenetic protein 8 (*bmp8*), progesterone receptor (*pgr*), sterol 26-hydroxylase (*cyp27a1*), and so on were highly expressed in the testes. However, genes such as aromatase (*cyp19a1a*), zona pellucida sperm-binding protein 3 (*zp3*), zona pellucida sperm-binding protein 4 (*zp4*), forkhead box protein L2 (*foxl2*), bone morphogenetic protein 1 (*bmp1*), luteinizing hormone receptor (lhr), activin receptor type-2B (*acvr2B*), inhibin beta (*inhb*), growth/differentiation factor 3 (*gdf3*), and so on were mainly expressed in the ovaries (Table 4).

### 3.4. Validation of DEGs by qRT-PCR

**Figure 6:**
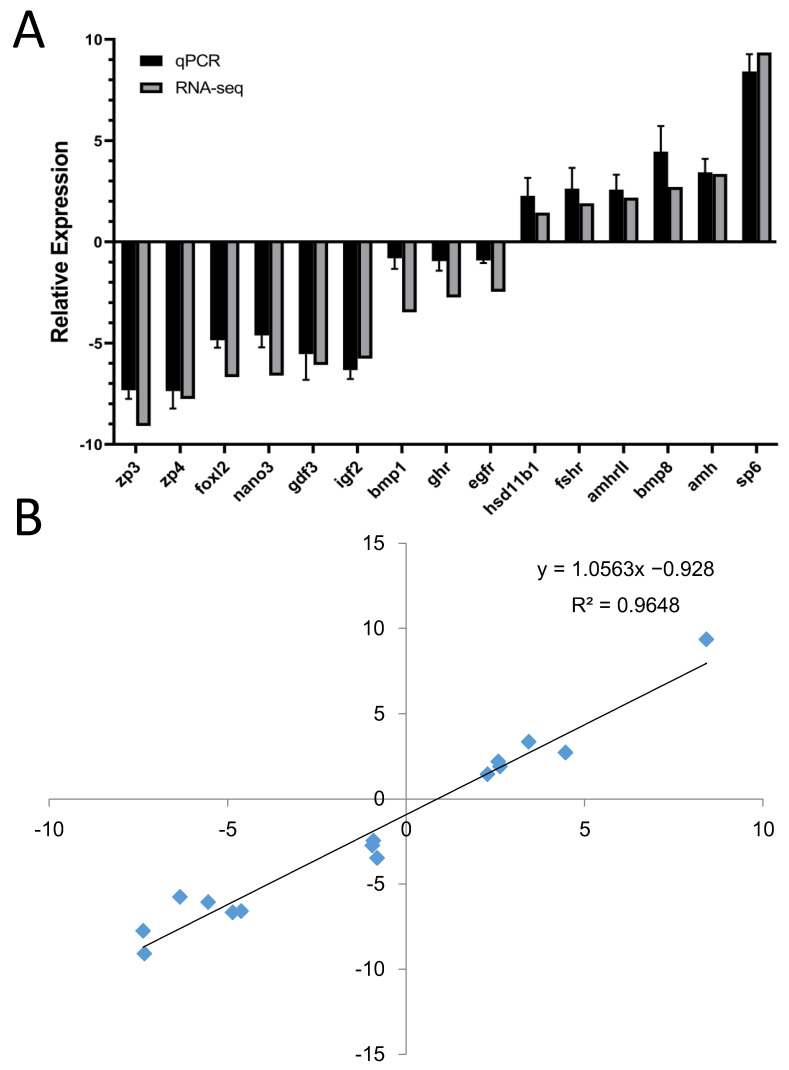
Validation of expression of six testis-biased and nine ovary-biased genes by qRT-PCR (**A**). The simple linear relationship of the transcriptome and qRT-PCR data (**B**).

To validate the expression profiles of the DEGs, nine female-biased and six male-biased genes were separately chosen for qRT-PCR analysis. The results showed that the expression of all DEGs validated by qRT-PCR was completely consistent with the results from the transcriptome data (Figure 6). The expression of DEGs including *amh*, *amhrII*, *cyp27a1*, *bmp8*, and *sp6* was male-biased, while the expression of DEGs such as *zp4*, *zp3*, *foxl2*, *nanos3*, *gdf3*, *igf2*, *bmp1*, and *egfr* was female-biased (Figure 6A). Moreover, a simple linear relationship revealed that the gene expression was highly consistent between the transcriptome and qRT-PCR data (Figure 6B).

## 4. Discussion

Understanding and mastering the expression patterns of reproduction-related genes is important for the analysis of gonad development and reproduction regulation. The rabbitfish is very popular with consumers in southeast China. However, its period of sexual maturity is very short, which limits the development of aquaculture. Thus, identifying its reproduction-related genes and mastering their expression pattern are critically important to realizing better management of reproduction activity. Here, for the first time, we carried out a comparative transcriptome analysis on testes and ovaries of rabbitfish and identified many reproduction-related genes.

As in other fish species [29,30], traditional sex-biased genes, such as *foxl2* and *cyp19a1a*, displayed ovary-biased expression in rabbitfish. In many fish species, knockout of *foxl2* or *cyp19a1a* reduces the level of E2 and further induces female-to-male sex reversal [29,30]. Thus, high expression of these genes in the ovaries suggests they might also play an important role in ovary development in rabbitfish. Meanwhile, *amh* and *amhrII* displayed testis-biased expression in rabbitfish. Previous studies demonstrated that after knocking out *amh* or *amhrII* in Nile tilapia, genetically male fish would develop into phenotypically female fish [31]. Thus, *amh* and *amhrII* might also play an important role in testis determination and development in rabbitfish. In addition, researchers also found that knocking out *amh* or *amhrII* in female fish caused ovarian termination [32]. Similar to results in *Coreoperca whiteheadi* [18] and *S. hollandi* [17], *zp3* and *zp4* also displayed ovary-biased expression in rabbitfish, indicating that they might also be essential for ovary development as a consequence of mediating the combination of sperm and ovum [17].

In addition to *amh* and *amhrII*, we also identified some additional members of the TGF-β family. Previous studies revealed that *bmp8* is mainly expressed in male germ cells and takes part in spermatogenesis by supporting the proliferation and survival of germ cells [33,34]. Mutation of a functional *bmp8* gene caused varying degrees of germ cell degeneration and defects in the initiation and maintenance of spermatogenesis [33,34]. Thus, high expression of *bmp8* in the testes suggests it might also play an important role in spermatogenesis in rabbitfish. However, different from *bmp8*, *bmp1* was mainly expressed in the ovaries of the rabbitfish. And a previous study also suggested that *bmp1* is expressed throughout porcine ovarian follicle development and promotes oocyte maturation in in vitro cultures [35]. It seems that *bmp1* also participates in the process of ovary maturation in rabbitfish. Similar to results in *S. hollandi* [17] and *C. whiteheadi* [18], we also found that *gdf3* was highly expressed in the ovaries of the rabbitfish. However, *gdf3* has been demonstrated to be required for mesendoderm formation and dorsal–ventral patterning, and mutation of *gdf3* causes abnormal development of zebrafish embryos [36]. Thus, whether *gdf3* participates in ovary development needs to be studied further. In addition, we also found that the expression of *inhb* was ovary-biased in rabbitfish. In mammals, inhibins including inhibin A and inhibin B have many regulatory effects on the ovaries, such as steroidogenesis, follicle development, and maturation [37]. Inhibin A and inhibin B could suppress FSH release and locally enhance follicle development [38]. The high expression of *inhb* in the ovaries also suggested it might be involved in ovary development in rabbitfish.

The sox gene family is another supergene family that plays a critical role in sex determination and reproduction regulation. Takehana et al. found a male-specific *sox3* gene, *sox3Y*, in *Oryzias dancena*, and found it could upregulate the expression of *gsdf* and other genes that further induced gonads to develop into testes [39]. In addition, a previous study also found that the *sox5* gene was involved in testis formation in *Oryzias latipes*. The main sex-determining gene was *dmy*, and *sox5* could downregulate *dmy* activity by binding to its promoter [40]. In addition, some members of the sox gene family also participate in ovary development. In this study, we found that the expression of *sox2* was ovary-biased in rabbitfish, but in *C. whiteheadi*, the expression of *sox2* was testis-biased [18]. However, previous research also found that *sox2* was moderately expressed in the testes and ovaries of *Larimichthys crocea* without a difference in expression [41,42]. Thus, it seems that *sox2* is involved in both testis and ovary development, but its function in rabbitfish reproduction is less clear. Meanwhile, the expression of *sox17* was ovary-biased in rabbitfish. Similarly, in *Dicentrarchus labrax*, high expression of *sox17* was also found in females, and its expression was closely correlated with the gonadal aromatase level, which indicated that *sox17* plays an important role in the development of the ovaries [43]. Also, ovary-biased expression was found for *sox6* and *sox11* in rabbitfish. These genes are also highly expressed in the gonads of female Chinese soft-shelled turtles [44]. Sox family genes participate in the process of testis and ovary development.

The wnt signaling pathway plays important roles in various physiological processes, especially in male and female gonad development [45]. In a comparative transcriptome analysis of female and male gonads, *wnt2*, *wnt3*, and *wnt5* were highly expressed in the testes, while *wnt8* was mainly expressed in the ovaries of *S. hollandi* [17]. However, in rabbitfish, we found that the expression of *wnt2b*, *wnt9b*, and *wnt16* was ovary-biased. A previous study also found that higher expression of *wnt2b* was detected in developing ovaries in turtles and humans [46]. Meanwhile, in mice, *wnt2b* was expressed in whole ovaries on days 0–21 postpartum [47]. Thus, it seems that *wnt2b* not only participates in ovary development but also plays a role in the process of postpartum repair. In rainbow trout (*Oncorhynchus mykiss*), dimorphic expression of *wnt9b* started during ovarian differentiation at the molecular level, and the expression of *wnt9b* increased in the ovaries after gonad differentiation at the histological level [45]. In fact, mutation of *wnt9b* in female mice caused them to lose their uteruses and upper vaginas, but their ovaries were normal [48]. Thus, the high expression of *wnt9b* in the ovaries of the rabbitfish suggests it also plays an important role in ovary development and maturation. For *wnt16*, it is mainly involved in regulating bone size in mice [49], with low expression detected in the oocytes of primordial follicles [50]. Whether it participates in ovary development needs to be studied further. As we found many candidate reproduction-related rabbitfish genes, further functional studies need to be carried out.

## 5. Conclusions

Based on Illumina sequencing technology, for the first time, we analyzed and compared gonad transcriptome data from female and male *S. oramin*. Many functional genes were successfully assembled and annotated. And through comparative transcriptome analysis of female and male gonads, a large number of DEGs participating in gonadal development and differentiation were identified, such as the TGF-β family, the sox gene family, and several wnt genes. Finally, the expression pattern of the DEGs was also validated by qRT-PCR. Generally, these data may contribute to further research on gonad development and reproduction regulation in *S. oramin*.

## Figures and Tables

**Table 1:** Primers used for qRT-PCR.

Primer ID	Primer Sequences
actinF	AATCGTGCGTGACATCAAG
actinR	GGAAGGAAGGCTGGAAGA
amhF	GTTTCCTGACCGAGGTCATGCC
amhR	AGCTCCGCCAGAAGCGTTTG
amhrIIF	TTTACTTCTCACGTGCCTCATG
amhrIIR	CAGCAACTGCTCCAGTTCAATAT
bmp1F	CGACTCCAGGTCACCGTATCAAA
bmp1R	AAACGCATGTCTCGCCCATC
bmp8F	ACTGGACCATAAATACCCCAAAAAG
bmp8R	TCCTGAAGAAGCACCGCAAC
egfr3F	ATGGTGAAATGCTGGATGATAGATG
egfr3R	TAGGGTCCGAGGGACTTGGT
foxl2F	AGGGTTGGCAGAACAGTATCAGG
foxl2R	GAAATGCGTCGGTGGAGGTC
fshF	TCAAACCATCGCTGAGATACGG
fshR	CCAACTCACTGGAACCCACAAA
ghr1F	AGCCACAGAGCCAGCAGACAA
ghr1R	GCCCACAATCCCAAAAAATAGG
hsd11b1F	GAGGGAGAAGGTCTTACAACAGG
hsd11b1R	TTGAGAATGAGATAATCCAACCC
igf2F	ACCTGTTCATCCCGGCGCTT
igf2R	TCCTCTGCTTGGCATCCATCC
nano3F	CACTCGCCACTGGTACTCAATTACTAAT
nano3R	GCGGGACGTGATGGAAGCTT
sp6F	CTGGCAGAGATGGTGGTGGA
sp6R	TGGACAGCTCAGGCGTGTGT
zp3F	GCCTTCAGGTTCCACCAGGAC
zp3R	GCCACCCATCTGTTGGTTTCG
ZP4F	TTGAGTGGTTGCTGGTGGTCC
ZP4R	CAGGTGTTTAGCGCTTTGTGG

**Table 2:** Statistical information related to the gonad transcriptome.

Sample ID	Reads (#)	Base (nt)	GC (%)	Q20 (%)	Q30 (%)
F1	61,675,162	9,251,274,300	51.69	98.39; 96.97	95.05; 91.47
F2	57,693,072	8,653,960,800	51.42	98.61; 97.81	95.76; 93.38
F3	48,472,486	7,270,872,900	51.99	98.56; 97.96	95.58; 93.81
M1	46,836,218	7,025,432,700	51.35	98.37; 97.31	95.02; 92.29
M2	48,159,824	7,223,973,600	51.52	98.54; 97.54	95.50; 92.74
M3	48,686,820	7,303,023,000	50.86	98.41; 97.50	95.21; 92.76

SampleID: sample name; Reads: the total number of sequences; Bases: the total number of sequencing bases; Q20 (%): the percentage of bases with a correct base recognition rate of more than 99%; Q30 (%): the percentage of bases with a correct base recognition rate of more than 99.9%; GC Content (%): the number of G + C bases as a percentage of the total number of bases.

**Table 3:** Information on the assembly of the gonad transcriptome of F1.

	Transcript	Unigene
Seqs Num (#):	71,435	47,070
Total length (nt):	108,866,991	54,799,987
Average length (nt):	1524	1164
Max length (nt):	23,938	23,938
Min length (nt):	189	189
GC:	49.67%	48.82%
N50:	2875	2488

**Table 4:** Information related to sex-related differentially expressed genes.

Unigene ID	log_2_ Fold Change (Testes vs. Ovaries)	*p*-Value (Testes vs. Ovaries)	FDR (Testes vs. Ovaries)	Nr Annotation	Gene Name
unigene020497	3.352	5.35 × 10^−11^	4.19 × 10^−10^	anti-Mullerian hormone	*amh*
unigene033316	2.184	1.97 × 10^−05^	5.86 × 10^−05^	anti-Mullerian hormone type-2 receptor	*amhrII*
unigene038440	2.715	1.09 × 10^−12^	1.12 × 10^−11^	bone morphogenetic protein 8	*bmp8*
unigene023167	3.447	1.23 × 10^−08^	6.52 × 10^−08^	sterol 26-hydroxylase	*cyp27a1*
unigene033805	2.154	5.47 × 10^−05^	1.50 × 10^−04^	progesterone receptor	*pgr*
unigene043282	6.890	2.77 × 10^−08^	1.38 × 10^−07^	sperm-associated antigen 17	*sp17*
unigene035428	9.357	4.66 × 10^−12^	4.29 × 10^−11^	sperm-associated antigen 6	*sp6*
unigene041677	−3.473	2.39 × 10^−07^	1.01 × 10^−06^	bone morphogenetic protein 1	*bmp1*
unigene027476	−2.687	4.49 × 10^−06^	1.51 × 10^−05^	activin receptor type-2B	*acvr2B*
unigene001102	−3.171	2.34 × 10^−02^	3.76 × 10^−02^	aromatase	*cyp19a1a*
unigene018423	−2.467	2.83 × 10^−11^	2.32 × 10^−10^	epidermal growth factor receptor	*egfr*
unigene000002	−3.255	1.18 × 10^−04^	3.03 × 10^−04^	fibroblast growth factor 13	*fgf13*
unigene035775	−2.514	1.05 × 10^−03^	2.25 × 10^−03^	fibroblast growth factor 16	*fgf16*
unigene008560	−3.554	1.87 × 10^−05^	5.59 × 10^−05^	fibroblast growth factor 20	*fgf20*
unigene011482	−2.568	6.69 × 10^−11^	5.14 × 10^−10^	fibroblast growth factor receptor 1	*fgfr1*
unigene019550	−3.799	8.28 × 10^−08^	3.79 × 10^−07^	forkhead box protein B1	*foxb1*
unigene040133	−6.681	8.14 × 10^−18^	1.62 × 10^−16^	forkhead box protein L2	*foxl2*
unigene021398	−6.080	1.31 × 10^−40^	1.34 × 10^−38^	growth/differentiation factor 3	*gdf3*
unigene043259	−2.742	1.04 × 10^−07^	4.67 × 10^−07^	growth hormone receptor	*ghr*
unigene016245	−5.765	3.13 × 10^−06^	1.08 × 10^−05^	insulin-like growth factor 2 mRNA-binding protein 3	*igfbp2*
unigene015144	−2.828	7.16 × 10^−05^	1.91 × 10^−04^	inhibin beta	*inhb*
unigene032568	−2.325	1.83 × 10^−03^	3.75 × 10^−03^	luteinizing hormone receptor	*lhr*
unigene019625	−6.603	6.28 × 10^−33^	3.91 × 10^−31^	nanos homolog 3	*nano3*
unigene027321	−2.135	1.08 × 10^−02^	1.88 × 10^−02^	steroidogenic factor 1	*nr5a1*
unigene042213	−3.339	6.49 × 10^−13^	6.85 × 10^−12^	mothers against decapentaplegic homolog 7	*smad7*
unigene032006	−3.884	4.82 × 10^−02^	7.17 × 10^−02^	Transcription factor sox11	*sox11*
unigene033133	−2.935	1.03 × 10^−11^	8.94 × 10^−11^	Transcription factor SOX-13	*sox13*
unigene010073	−2.919	2.73 × 10^−05^	7.92 × 10^−05^	transcription factor SOX-17	*sox17*
unigene031183	−3.234	1.32 × 10^−05^	4.08 × 10^−05^	Transcription factor Sox-2	*sox2*
unigene046385	−4.074	5.47 × 10^−10^	3.63 × 10^−09^	transcription factor SOX-6	*sox6*
unigene037102	−3.319	6.68 × 10^−20^	1.65 × 10^−18^	stAR-related lipid transfer protein 13	*star13*
unigene010465	−2.031	1.45 × 10^−09^	8.97 × 10^−09^	transforming growth factor beta regulator 1	*tgfb1*
unigene031121	−4.284	1.50 × 10^−20^	3.93 × 10^−19^	vascular endothelial growth factor D	*vegfD*
unigene034851	−5.654	9.65 × 10^−15^	1.33 × 10^−13^	protein Wnt-16	*wnt16*
unigene011231	−2.496	5.62 × 10^−06^	1.85 × 10^−05^	protein Wnt-2b	*wnt2b*
unigene000882	−2.937	4.66 × 10^−06^	1.56 × 10^−05^	protein Wnt-9b	*wnt9b*
unigene017893	−3.974	1.59 × 10^−28^	7.75 × 10^−27^	Wilms tumor protein 1	*WT1*
unigene046736	−9.090	2.65 × 10^−78^	3.73 × 10^−75^	zona pellucida sperm-binding protein 3	*zp3*
unigene014003	−7.758	1.10 × 10^−19^	2.66 × 10^−18^	zona pellucida sperm-binding protein 4	*zp4*

## Data Availability

The raw data are available from the SRA (http://www.ncbi.nlm.nih.gov/sra/, accessed on 12 October 2021) data repository (accession number: PRJNA1025886).
